# Stability of television viewing and electronic game/computer use in a prospective cohort study of Australian children: relationship with body mass index

**DOI:** 10.1186/1479-5868-4-60

**Published:** 2007-11-18

**Authors:** Kylie Hesketh, Melissa Wake, Melissa Graham, Elizabeth Waters

**Affiliations:** 1 Deakin University, 221 Burwood Hwy, Burwood Vic 3125, Australia; 2 Murdoch Childrens Research Institute, Flemington Rd, Parkville 3052, Australia

## Abstract

**Background:**

While much cross-sectional data is available, there have been few longitudinal investigations of patterns of electronic media use in children. Further, the possibility of a bi-directional relationship between electronic media use and body mass index in children has not been considered. This study aimed to describe longitudinal patterns of television viewing and electronic game/computer use, and investigate relationships with body mass index (BMI).

**Methods:**

This prospective cohort study was conducted in elementary schools in Victoria, Australia. 1278 children aged 5–10 years at baseline and 8–13 years at follow-up had their BMI calculated, from measured height and weight, and transformed to z-scores based on US 2000 growth data. Weight status (non-overweight, overweight and obese) was based on international BMI cut-off points. Weekly television viewing and electronic game/computer use were reported by parents, these were summed to generate total weekly screen time. Children were classified as meeting electronic media use guidelines if their total screen time was ≤14 hrs/wk.

**Results:**

Electronic media use increased over the course of the study; 40% met guidelines at baseline but only 18% three years later. Television viewing and electronic game/computer use tracked moderately and total screen time was positively associated with adiposity cross-sectionally. While weaker relationships with adiposity were observed longitudinally, baseline z-BMI and weight status were positively associated with follow-up screen time and baseline screen time was positively associated with z-BMI and weight status at follow-up. Children who did not meet guidelines at baseline had significantly higher z-BMI and were more likely to be classified as overweight/obese at follow-up.

**Conclusion:**

Electronic media use in Australian elementary school children is high, increases with age and tracks over time. There appears to be a bi-directional association suggesting that interventions targeting reductions in either screen time or adiposity may have a positive effect on both screen time and adiposity.

## Background

High levels of electronic media use during childhood have detrimental effects on child[1] and adult [2] health, including overweight [2,3]. Despite this, children's use of such media appears to have increased over recent decades [4]. Therefore, several countries now recommend a maximum of two hours per day electronic media use in their children's physical activity guidelines [5,6]. Television is the form of electronic media used most by children [7].

Children's television viewing patterns have been well described in cross-sectional studies. Girls and boys appear to watch similar amounts of television [7], and older children spend more time in front of the television than their younger peers [8-12]. A weak positive association between television viewing and adiposity in children is commonly reported [4,13].

Patterns of other electronic media use in children have received less attention, perhaps because children spend less time on these activities [9,14,15]. Boys tend to spend more time than girls using electronic games and computers [9,16,17], and usage appears to increase with age in both genders [9,17]. Electronic game use [14,16] and total sedentary time [15,17] have also been reported to be associated with adiposity in children.

To date, most investigations of electronic media use patterns in elementary school children have come from cross-sectional studies. Longitudinal descriptions of electronic media use and changes in usage over time in individual children have been scarce. The available data suggests that television viewing tracks quite strongly within the middle childhood period (correlations range from r = 0.46 to r = 0.73) [18-20], and higher television viewing times at one point in childhood appear to predict higher viewing later in childhood [8,21]. Electronic game use appears to track less strongly than television viewing time [18].

Longitudinal associations between electronic media use and adiposity in childhood have been investigated in different ways in different studies, making comparisons difficult. Unlike the consistent weak positive associations reported in cross-sectional studies for all forms of electronic media use [13], findings are more equivocal as to whether all forms of electronic media use predict increasing adiposity. Positive associations have been reported between television viewing and later adiposity [18,21-26] or increased adiposity [18,22,23] in most, but not all [27] studies. Conversely, electronic games have generally been found not to be associated with adiposity longitudinally [18,27], though some studies have reported an association for girls [27] or boys only [20]. Total screen time may have a positive [20,28] or no association with adiposity in childhood [20]. Despite these conflicting findings, the larger-scale and more recent studies generally suggest a positive association between electronic media use and adiposity [29].

This paper aims to (1) describe longitudinal patterns of television viewing and electronic game/computer use in a large population-ascertained sample of Australian children as they move from the early to late elementary school years and (2) investigate relationships between television and electronic game/computer use and BMI trajectories.

## Methods

### Sample

This cohort was derived from the Health of Young Victorians Study (HOYVS), conducted between September and December 1997. At baseline, HOYVS employed a two-stage stratified random sampling design to draw a sample of children representative of the state of Victoria, Australia. Twenty-four elementary schools were selected, with a probability proportional to school size (total enrolment), to be representative of the school sectors (government, Catholic and independent) and the geographic distribution of the state. At each school, one class at each year level (preparatory to grade six) was randomly selected for the study except where total school enrolment did not exceed 240 students, in which case the entire school was included. Children in grades preparatory to three (aged 5–10 years) in the baseline study were included in the follow-up study, conducted between October 2000 and June 2001 when children were aged 8–13 years. The average time between assessments was 3.2 years (SD = 0.2 years).

Both studies received approval from the Royal Children's Hospital Ethics in Human Research Committee and the relevant education authorities, and parents provided written informed consent in both waves.

### Measures

Children had their height and weight measured by trained researchers at baseline and follow-up. Investigation of anthropometric reliability found no evidence of systematic bias for intra- or inter-rater comparisons. Body mass index (BMI) was calculated (weight (kg)/height (m)^2^) and transformed into standardised (z) scores based on gender and exact age using the US Centers for Disease Control and Prevention 2000 growth chart data [30]. Children were also classified in one of three BMI categories (non-overweight, overweight and obese) using international gender- and age-specific cut-off points [31].

At baseline and follow-up, parents completed a self-administered questionnaire reporting on their child's television viewing and electronic game/computer use for an average school day and average non-school day. At baseline, six-point ordinal response scales were used, with duration taken to be the midpoint of each category ('none', 'less than 1 hour', '1–2 hours', '3–4 hours' and '5–6 hours', coded as 0, 0.5, 1.5, 3.5 and 5.5 hours respectively) except the '7 or more hours' category (coded as 7.5 h). At follow-up, open ended continuous response scales ('___ hours') were used. Weekly scores were calculated by summing hours reported for an average school day (multiplied by five) and average non-school day (multiplied by two). Weekly television viewing time and weekly electronic game/computer use were summed to create a total screen time variable. Change in television time, electronic game/computer use time and total screen time scores were generated by subtracting weekly scores at baseline from weekly scores at follow-up. In addition, children were classified as meeting electronic media use guidelines if their total screen time did not exceed 14 hours per week. Parents provided standard demographic information.

### Analyses

Baseline characteristics of responders and non-responders were compared using independent samples t-tests for continuous variables and chi-square statistics for categorical variables. Differences in outcome variables by gender were assessed using independent samples t-tests and differences by age were assessed using linear regression analyses. Pearson's correlations assessed the relationship between outcome variables at baseline and follow-up. Differences in the proportions of children meeting guidelines for electronic media use by gender were assessed using the chi-square statistic, and by age using logistic regression analyses.

Cross-sectional relationships between screen time and BMI category or z-BMI were assessed using linear regression, adjusted for age. Cross-sectional relationships between proportions of children meeting electronic media use guidelines and BMI category or z-BMI were assessed using logistic regression, adjusted for age.

Only children with complete BMI and screen time data at both time points were included in analyses of temporal relationships. For analyses of temporal relationships BMI categories were collapsed to non-overweight and overweight/obese. Pearson's correlation coefficients were used to assess the relationship between z-BMI at baseline and follow-up and between total screen time at baseline and follow-up. Independent samples t-test statistics compared differences in mean z-BMI between children who did and did not meet electronic media use guidelines. Pearson's chi-square statistics assessed differences in the proportions of children classified as overweight/obese who did and did not meet electronic media use guidelines. Longitudinal relationships between z-BMI or BMI category and screen time were assessed using linear regression, logistic regression and analysis of variance analyses, as appropriate, each adjusting for child age. Longitudinal relationships between z-BMI or BMI category and electronic media use guidelines were assessed using Pearson's chi-square statistic or independent samples t-test analyses as appropriate.

## Results

### Sample

Of the 1943 children who participated in the baseline study, 204 were unable to be located and 170 declined to participate at follow-up. Thus 1569 children provided data at follow-up, a retention rate of 81% of the cohort. Baseline characteristics of the 1569 responders and 374 children lost to follow-up (non-responders) were compared. Responders and non-responders were similar in terms of gender, age, maternal and paternal education levels, weekly television viewing, weekly electronic game/computer use and total screen time. However, the mean BMI of non-responders was higher than that of responders (17.5 versus 16.9 kg/m^2^; p = 0.02), and more non-responders were classified as overweight or obese (18.4% versus 15.1% overweight and 9.5% versus 4.6% obese; p < 0.001) at baseline. In addition, non-responders were more likely to live in urban areas (66.6% versus 61.2%; p = 0.07) and to have parents born in a non-English speaking country (20.1% versus 12.8%; p < 0.001).

Parent questionnaire data was available at both time points for 1278 children, who comprise the sample reported here. Of these, 1163 children also provided anthropometric data at both time points and are included in analyses including BMI or weight status.

### Screen time

Table 1 summarises children's screen time at baseline and follow-up. Mean television viewing rose by 1.2 hrs/wk (SD = 7.6) over the course of the study. Girls showed greater increases in television time than boys (p = 0.06). Baseline and follow-up television time were moderately correlated (r = 0.48). Although television time rose with age across both the baseline (p = 0.007) and follow-up (p < 0.001) data, there was no difference by age in television time *change *over the three years. Boys electronic games/computer use was higher than girls (p < 0.001 at both time points), and usage rose with age (p < 0.001 at both time points). Baseline and follow-up electronic game/computer use were moderately correlated (r = 0.34). Usage rose substantially over the three years (by 4.6 hrs/wk (SD = 6.7)), with greater increases for boys than girls (p < 0.001) but no differences by age.

**Table 1:** Age, screen time and anthropometric characteristics of the sample (n = 1278; 630 boys, 648 girls).

	**Baseline**			**Follow-up**		
		
**Characteristic**	**Total sample**	**Males**	**Females**	**Total sample**	**Males**	**Females**
Age (yrs) [m (SD)]	7.6 (1.2)	7.7 (1.2)	7.5 (1.2)	10.7 (1.2)	10.8 (1.2)	10.7 (1.2)
TV viewing (hrs/wk) [m (SD)]	14.0 (7.6)	14.4 (7.8)	13.7 (7.5)	15.2 (7.1)	15.2 (7.3)	15.2 (6.8)
Games/computer (hrs/wk) [m (SD)]	3.9 (4.1)	4.9 (4.5)	3.0 (3.3)	8.5 (6.9)	10.6 (7.4)	6.3 (5.6)
Total screen time (hrs/wk) [m (SD)]	18.0 (9.2)	19.3 (9.6)	16.7 (8.6)	23.7 (10.7)	25.8 (11.5)	21.6 (9.3)
Meet guidelines [n (%)]*	509 (40)	220 (35)	289 (45)	226 (18)	73 (12)	153 (24)
BMI z-score [m (SD)]	0.4 (0.9)	0.5 (0.8)	0.4 (0.9)	0.4 (0.9)	0.4 (0.9)	0.3 (0.9)
BMI category [n (%)]:						
Not overweight	959 (81)	478 (82)	481 (79)	953 (76)	483 (78)	470 (74)
Overweight	178 (15)	83 (14)	95 (16)	246 (20)	112 (18)	134 (21)
Obese	51 (4)	22 (4)	29 (5)	52 (4)	24 (4)	28 (4)

*Australian Physical Activity Recommendations for children indicate no more than 2 hrs per day should be spent engaged in electronic media use [5].

Similar changes were observed for total screen time, which increased by an average of 5.7 hrs/wk (SD = 10.4) over three years, more so for boys than girls (p = 0.006). Total screen time at baseline and follow-up were moderately correlated (r = 0.46). At baseline, 40% of children met the Australian guideline of two hours or less of electronic media use per day (≤14 hrs/wk) but only 18% met this guideline at follow-up. More girls than boys met the guideline (p < 0.001 at both time points) and the odds of meeting the guideline decreased with age (p = 0.001 at baseline; p < 0.001 at follow-up).

### Cross sectional relationships between screen time and body mass index

Table 2 shows cross-sectional relationships between screen time and BMI category. At both time points, mean hours of television viewing increased in a step-wise fashion across BMI categories for girls (p = 0.02 and p < 0.001 for overweight and obese respectively at baseline; p = 0.001 and p = 0.003 at follow-up). However, compared to non-overweight boys, overweight (p = 0.03 and p = 0.001 for baseline and follow-up respectively) but not obese (p = 0.39 and p = 0.43) boys had higher mean weekly television time. At baseline, obese girls spent more time using electronic games/computer than non-overweight girls (p = 0.003) but no differences by BMI categories were observed for boys. At follow-up, overweight boys spent 1.6 times more hours per week using electronic games/computer (p = 0.04) and obese boys four times more hours (p = 0.007) than non-overweight boys. Girls *total *screen time increased across BMI categories at both baseline and follow-up, and boys at follow-up only.

**Table 2:** Cross-sectional analysis of differences in screen time across BMI categories.

**Activity**	**BMI category**	**Total sample**		**Males**		**Females**	
		
		m (SD) *or *%	β *or *OR (95% CI)^†^	m (SD) *or *%	β *or *OR (95% CI)^†^	m (SD) *or *%	β *or *OR (95% CI)^†^
**Baseline**							
TV viewing (hrs/wk)	Non-overweight	13.5 (7.4)	-	13.9 (7.6)	-	13.1 (7.1)	-
	Overweight	15.6 (8.5)	1.9 (0.7, 3.1)	16.4 (8.5)	2.0 (0.2, 3.9)	15.0 (8.5)	1.9 (0.3, 3.5)
	Obese	16.7 (7.8)	3.4 (1.3, 5.5)	15.0 (8.1)	1.5 (-1.9, 4.9)	18.0 (7.4)	5.0 (2.2, 7.7)
Games/computer (hrs/wk)	Non-overweight	3.9 (4.1)	-	4.9 (4.6)	-	2.9 (3.2)	-
	Overweight	3.5 (3.8)	-0.3 (-0.9, 0.3)	4.3 (4.2)	-0.6 (-1.6, 0.5)	2.9 (3.3)	0.0 (-0.7, 0.8)
	Obese	5.4 (4.6)	1.5 (0.4, 2.7)	6.3 (5.8)	1.4 (-0.6, 3.3)	4.7 (3.5)	1.9 (0.6, 3.1)
Total screen time (hrs/wk)	Non-overweight	17.4 (8.9)	-	18.9 (9.5)	-	16.0 (8.1)	-
	Overweight	19.2 (10.2)	1.6 (0.2, 3.1)	20.6 (10.7)	1.5 (-0.8, 3.7)	17.9 (9.7)	2.0 (0.1, 3.8)
	Obese	22.1 (8.9)	4.9 (2.4, 7.5)	21.3 (9.4)	2.9 (-1.3, 7.0)	22.7 (8.6)	6.8 (3.7, 10.0)
Meet guidelines*	Non-overweight	42.4%	-	37.6%	-	47.1%	-
	Overweight	34.5%	0.7 (0.5, 1.0)	27.7%	0.7 (0.4, 1.1)	40.4%	0.8 (0.5, 1.2)
	Obese	23.5%	0.4 (0.2, 0.8)	27.3%	0.5 (0.2, 1.5)	20.7%	0.3 (0.1, 0.7)
**Follow-up**							
TV viewing (hrs/wk)	Non-overweight	14.6 (6.7)	-	14.6 (6.8)	-	14.6 (6.6)	-
	Overweight	17.0 (7.9)	2.4 (1.4, 3.3)	17.2 (8.9)	2.6 (1.1, 4.1)	16.8 (7.1)	2.2 (0.9, 3.4)
	Obese	17.1 (6.4)	2.6 (0.6, 4.5)	15.8 (6.3)	1.2 (-1.8, 4.2)	18.2 (6.4)	3.8 (1.3, 6.3)
Games/computer (hrs/wk)	Non-overweight	8.2 (6.5)	-	10.0 (6.9)	-	6.3 (5.5)	-
	Overweight	8.8 (7.4)	0.6 (-0.3,1.5)	11.7 (8.3)	1.6 (0.1, 3.1)	6.4 (5.6)	0.2 (-0.9, 1.2)
	Obese	10.7 (7.6)	2.7 (0.8, 4.5)	14.1 (6.9)	4.0 (1.1, 7.0)	7.9 (7.1)	1.8 (-0.3, 3.9)
Total screen time (hrs/wk)	Non-overweight	22.8 (10.0)	-	24.6 (10.5)	-	20.9 (9.1)	-
	Overweight	25.8 (11.9)	3.0 (1.5, 4.4)	28.9 (13.7)	4.2 (1.9, 6.5)	23.2 (9.3)	2.3 (0.6, 4.1)
	Obese	27.8 (10.6)	5.2 (2.4, 8.1)	29.9 (9.5)	5.2 (0.7, 9.8)	26.0 (11.3)	5.6 (2.1, 9.1)
Meet guidelines*	Non-overweight	19.0%	-	12.7%	-	25.5%	-
	Overweight	14.2%	0.7 (0.5, 1.1)	9.8%	0.8 (0.4, 1.5)	17.9%	0.6 (0.4, 1.0)
	Obese	11.5%	0.5 (0.2, 1.3)	0%	††	21.4%	0.7 (0.3, 1.9)

^†^Linear regression (or logistic regression for proportion meeting guidelines) analyses adjusted for age.

* Australian Physical Activity Recommendations for children indicate no more than 2 hrs/day should be spent engaged in electronic media use [5].

†† Odds ratio not calculated as no obese boy met guidelines at follow-up

Examining relationships between weekly screen time and z-BMI yielded stronger associations. At both baseline and follow-up, television viewing time increased with increasing z-BMI for boys (β = 1.17, 95% CI = 0.41, 1.94 and β = 0.72, 95% CI = 0.09, 1.35 respectively) and girls (β = 1.06, 95% CI = 0.38, 1.74 and β = 1.20, 95% CI = 0.64, 1.76). Similarly, a positive association was observed between electronic game/computer use and z-BMI for boys (β = 0.42, 95% CI = -0.02, 0.86 at baseline; β = 1.28, 95% CI = 0.66, 1.89 at follow-up) and girls (β = 0.34, 95% CI = 0.04, 0.64 and β = 0.34, 95% CI = -0.12, 0.80), although not all associations reached significance. Total screen time was strongly associated with z-BMI at both time points for boys (β = 1.61, 95% CI = 0.66, 2.55 and β = 1.99, 95% CI = 1.03, 2.96) and girls (β = 1.41, 95% CI = 0.64, 2.19 and β = 1.55, 95% CI = 0.78, 2.31). The proportion of children who met electronic media use guidelines decreased with increasing z-BMI at baseline and follow-up for both boys (OR = 0.78, 95% CI = 0.63, 0.97 and OR = 0.83, 95% CI = 0.63, 1.09) and girls (OR = 0.80, 95% CI = 0.66, 0.97 and OR = 0.78, 95% CI = 0.65, 0.96), although the association for boys at follow-up did not reach significance.

### Temporal relationships between screen time and body mass index

BMI and screen time data were available at both time points for 1151 children who form the sample for this section of the paper. Baseline and follow-up z-BMI were highly correlated (r = 0.84). Total screen time at baseline and follow-up were moderately correlated (r = 0.45). The vast majority (85%) of children remained in the same BMI category (non-overweight, overweight or obese) between baseline and follow-up and most (67%) remained in the same electronic media use guideline category (meet or do not meet guidelines). However, more than one quarter (28%) of all children were classified as meeting electronic media use guidelines at baseline but not follow-up. These findings were similar for boys and girls. Mean z-BMI and the proportion of children classified as overweight/obese was higher for children who did not meet electronic media use guidelines than for those who did (Table 3).

**Table 3:** Proportion of children meeting electronic media use guidelines^†^by weight status

Meet electronic media use guidelines^†^	Mean z-BMI		Proportion overweight/obese* (%)	
	
	Baseline	Follow-up	Baseline	Follow-up
Baseline				
Yes	0.34	0.23	14.8	19.4
No	0.49	0.45	21.7	26.8
p-value	0.003^a^	<0.001^a^	0.003^b^	0.004^b^
Follow-up				
Yes	0.34	0.17	14.7	18.1
No	0.45	0.40	19.9	25.0
p-value	0.09^a^	0.001^a^	0.09^b^	0.04^b^

*overweight & obese categories combined

^a ^t-test statistic; ^b ^chi-square statistic

^† ^Australian Physical Activity Recommendations for children indicate no more than 2 hrs/day should be spent engaged in electronic media use [5].

Baseline screen time predicted follow-up z-BMI (β = 0.02, 95% CI = 0.01, 0.02) and BMI category (non-overweight or overweight/obese; OR = 1.03, 95% CI = 1.02, 1.05) after adjustment for child age. This suggests that for every extra hour per week a child is engaged in screen-based activities, their odds of being overweight three years later increases by 3%. Children spending more time in screen-based activities and those failing to meet electronic media use guidelines at baseline tended to have higher z-BMIs and were more likely to be classified as overweight/obese at follow-up (Table 3). The same pattern of results was observed for girls and boys. While baseline screen time did not predict change in z-BMI, children who met electronic media use guidelines at baseline had greater declines in z-BMI over the course of the study (-0.10 versus -0.04, p = 0.04). However when stratified by gender this relationship was observed for girls only (-0.13 versus -0.03, p = 0.01).

An association was also found in the opposite direction. Children with higher z-BMIs and those classified as overweight/obese at baseline had higher follow-up screen time after adjusting for age (β = 1.59, 95% CI = 0.89, 2.29 and F = 8.1, p = 0.005). Children classified as overweight/obese at baseline had two hrs/wk more screen time at follow-up than non-overweight children (25.2 versus 23.0, p = 0.006); the difference was less than two hrs/wk for girls (22.9 versus 21.2, p = 0.08) and three hrs/wk for boys (27.9 versus 24.9, p = 0.01). Baseline z-BMI did not predict change in screen time and there was no difference in screen time change between children classified as non-overweight and overweight/obese at baseline. There was a tendency for girls whose screen time increased to also show increases in z-BMI (β = 1.44, 95% CI = -0.10, 2.99, p = 0.07), but this trend was not seen for boys (β = 0.65, 95% CI = -1.09, 2.34, p = 0.46).

## Discussion

Patterns of screen time (both television and electronic games/computer use) appear well-established by the early elementary school years and track quite strongly through middle childhood. Of concern, children's screen time increased with age and children became less likely to meet recommended guidelines as they got older. By late elementary school, less than 20% of this sample met the recommendation of spending less than two hours per day using electronic media. Previous research has established that adolescents spend more time in screen based activities than their younger counterparts [2], and have greater personal access to such media as they are more likely to have a television and computer in their bedroom [33]. Thus it appears likely that as the children from this study transition into adolescence, their screen time will increase and the numbers meeting recommended guidelines will further diminish.

Cross-sectional relationships between screen time and BMI were stronger than the relationships observed longitudinally. This trend was also observed in a meta-analysis [13] reporting a small but statistically significant relationship between television viewing and body fatness in children, with a larger effect size for cross-sectional than longitudinal studies, although the difference was not statistically significant. However, two studies [22,23] published since the meta-analysis report contrasting findings, with no association between BMI and television viewing at baseline, but a positive association longitudinally. Both of these recent studies involved small samples (<300 children between them) so their inclusion in the meta-analysis is unlikely to alter its conclusions.

Despite equivocal findings in the literature [8], screen time and BMI were clearly found to be related in this study. It has been suggested that a simple question about screen time (whether or not a child meets the ≤2 hours per day guideline) may be an effective screening tool for children at risk of overweight [32]. The findings of this study support such a suggestion, as children who failed to meet the guidelines were more likely to be overweight at that point in time as well as three years later.

The relationship between screen time and BMI was bi-directional: baseline screen time predicted higher follow-up BMI and baseline BMI predicted higher follow-up screen time. Previous studies have tended to focus on whether screen time predicts adiposity. The findings of this study are important as they suggest that interventions targeting either reduction in screen time or adiposity may have a positive effect on both screen time and overweight. Further research is required to confirm this bi-directional relationship.

This study is limited by its observational nature to be able to suggest directions of associations but not infer causality. The subjective measures of screen time employed may have resulted in underreporting of these behaviours by parents and such reporting bias may have differed by the weight status of the child. Yet such reporting bias is likely to have resulted in under-rather than over-estimation of the screen time behaviours and the association between screen time and adiposity. While the response scales for the screen time measures differed between baseline (categorical) and follow-up (continuous), both were converted to continuous scales to allow for comparative analysis. A higher proportion of overweight than non-overweight children were lost to follow-up. While this resulted in a smaller proportion of overweight children in the sample, it is unlikely to have influenced the relationships observed between BMI and screen time as the baseline screen time behaviours of responders and non-responders did not differ. The strengths of this study include its large sample size and population-based sampling design, and its high response and retention rates. The vast majority of previous studies of electronic media use have focused exclusively on television viewing. This study also included a measure of electronic game/computer use which was also found to be a prevalent behaviour.

## Conclusion

Television viewing and electronic game/computer use are prevalent pastimes for elementary school children which become more prevalent as the children get older. These pastimes appear to be associated with overweight and may represent a useful means of assessing the risk of overweight in clinical practice. The apparent bi-directional nature of the association between electronic media use and overweight suggests that interventions designed to reduce the prevalence of one may have a positive effect on both.

## Competing interests

The author(s) declare that they have no competing interests.

## Authors' contributions

KH participated in the design and coordination of the study, performed the statistical analyses and drafted the manuscript. MW participated in the design and coordination of the study and contributed to the development of the manuscript. MG contributed to the development of the manuscript. EW participated in the design and coordination of the study and contributed to the development of the manuscript. All authors read and approved the final manuscript.
